# A randomized controlled pilot study to evaluate the effect of an enteral formulation designed to improve gastrointestinal tolerance in the critically ill patient—the SPIRIT trial

**DOI:** 10.1186/s13054-017-1730-1

**Published:** 2017-06-10

**Authors:** Stephan M. Jakob, Lukas Bütikofer, David Berger, Michael Coslovsky, Jukka Takala

**Affiliations:** 1 Department of Intensive Care Medicine, Inselspital, Bern University Hospital, University of Bern, Bern, CH-3010 Switzerland; 2 CTU Bern, and Institute of Social and Preventive Medicine, University of Bern, Bern, Switzerland

**Keywords:** Diarrhea, Enteral nutrition, Intensive care, Critically ill

## Abstract

**Background:**

Diarrhea is frequent in patients in intensive care units (ICU) and is associated with discomfort and complications and may increase the length of stay and nursing workload.

**Methods:**

This was a prospective, double-blind, randomized, controlled single-center pilot study to assess the incidence and frequency of diarrhea and the respective effects of a modified enteral diet (intervention: Peptamen® AF, rich in proteins, medium chain triglycerides and fish oil) compared to a standard diet (control: Isosource® Energy) in 90 randomized adult patients (intervention, *n* = 46; control, *n* = 44) with an ICU stay ≥5 days and tube feeding ≥3 days. Tube feeding was initiated within 72 h of ICU admission and continued up to 10 days. The caloric goal was adjusted to needs by indirect calorimetry. Gastrointestinal function, nutritional intake, and nursing workload were recorded. Follow-up was until 28 days after randomization.

**Results:**

Median age was 63.3 (interquartile range (IQR) 51.0–73.2) years and Simplified Acute Physiology Score (SAPS) II was 61.0 (IQR 47.8–74). Time to reach caloric goal (intervention: 2.2 (0.8–3.7) days (median, IQR); control: 2.0 (1.3–2.7) days; *p* = 0.16), length of time on study nutrition (intervention: 5.0 (3.6–6.4) days; control: 7.0 (5.3–8.7) days; *p* = 0.26), and calorie intake (intervention: 18.0 (12.5–20.9) kcal/kg/day; control 19.7 (17.3–23.1) kcal/kg/day; *p* = 0.08) did not differ between groups, with a higher protein intake for Peptamen® group (1.13 (0.78–1.31) g/kg/day vs 0.80 (0.70–0.94); *p* < 0.001). No difference in diarrhea incidence (intervention group: 29 (64%); control group: 31 (70%); *p* = 0.652), use of fecal collectors (23 (51%) vs. 24 (55%); *p* = 0.83), or diarrhea-free days (161 (64%) vs 196 (68%); *p* = 0.65) was found. Nursing workload and cost for diarrhea care were not different between the groups. In a post-hoc analysis, adjusted for treatment group, age, sex, and SAPS II score, diarrhea was associated with length of mechanical ventilation (9.5 (6.0–13.1) vs. 3.9 (3.2–4.6) days; *p* = 0.006) and length of ICU stay (11.0 (8.9–13.1) vs. 5.0 (3.8–6.2) days; *p* = 0.001).

**Conclusions:**

In this pilot study, we found a high incidence of diarrhea, which was not attenuated by Peptamen® AF. Patients with diarrhea stayed longer in the ICU.

**Trial registration:**

ClinicalTrials.gov identifier, NCT01581957. Registered on 18 April 2012.

**Electronic supplementary material:**

The online version of this article (doi:10.1186/s13054-017-1730-1) contains supplementary material, which is available to authorized users.

## Background

Gastrointestinal dysfunction is common in critically ill patients with prolonged intensive care unit (ICU) stay [1], and diarrhea is one of the leading clinical symptoms [2–5]. The causes of diarrhea are multifactorial and include recent abdominal surgery, infection, decreased gastrointestinal perfusion, and antibiotics. In a prospective study in patients during the first 2 weeks of ICU stay, the combination of enteral nutrition covering >60% of the energy target and antibiotics or antifungal drugs increased the incidence of diarrhea [6].

The inability to absorb sufficient amounts of nutrition can increase muscle wasting and prolong recovery from critical illness [7]. Attempts to reduce diarrhea using fiber-enriched enteral formulas have been disappointing in ICU patients [8–11]. Diarrhea, independent of its reason, may predispose patients to a risk of malnutrition and development of decubitus ulcers. It causes substantial discomfort for patients and is likely to add to the workload of nurses and the cost of care.

There is a wide range of diarrhea incidence reported in recent randomized clinical studies (14% [6], 19–23% [12], 22–26% [13], 26–29% [14], and 33–92% [15]), partly related to patient population and varying diarrhea definition. Reports on diarrhea-related costs are scarce.

We performed a pilot study to test the effect of a new enteral formula on the frequency of diarrhea and gastrointestinal tolerance, and on all diarrhea-related costs in ICU long-stayers. The purpose of the study was also to gain information for the design of a future confirmatory trial. The tested formula has a high energy density in order to reduce the necessary feeding volume, enzymatically hydrolyzed whey protein to improve absorption [16], and its lipid fraction contains 50% medium chain triglycerides and fish oil due to their possible benefits in inflammatory states [17].

## Methods

This study was approved by the local Ethics Committee (KEK Bern, 060/12) and is registered at clinicalTrials.gov (NCT01581957). Informed consent was obtained from each patient or from a close relative. The study was designed as a prospective, double-blind, randomized, controlled single-center pilot study, where Peptamen® AF was compared in a 1:1 allocation to a standard formula (Isosource® Energy) with the same amount of caloric density. The allocation sequence was generated by an independent statistician not involved in the final analysis of the trial. It was based on computer generated random numbers in randomly varying blocks of four and six using the statistical software package Stata (StataCorp LP, College Station, TX, USA). Randomization was stratified by the presence or absence of diarrhea at the time of randomization. The study was conducted from 8 January 2013 (first patient included) until 29 August 2014 (last follow-up).

### Objectives

The primary objective was to evaluate diarrhea and other gastrointestinal symptoms in critically ill patients with prolonged ICU stay who are either fed with Peptamen® AF or Isosource® Energy. Secondary objectives were determination of inflammatory status, organ function, workload and cost, and safety of patients fed with either one of the two products. Accordingly, the primary outcomes were diarrhea-free days and number of diarrhea events per day during enteral nutrition administration in the ICU. Diarrhea was assessed according to the definition by Whelan et al. [18] from start of enteral nutrition until the end of enteral feeding or ICU discharge, whichever came first. Whelan assigned scores to three categories of stool amounts (<100 g, 100–200 g, >200 g) and four categories of stool consistencies (hard and formed, soft and formed, loose and unformed, liquid), ranging from 1 (<100 g, hard and formed) to 12 (>200 g, liquid). Diarrhea is defined as a score of 15 or more during 1 day. A list of the secondary outcomes is provided in Additional file 1.

### Inclusion and exclusion criteria

Inclusion criteria were medical and surgical ICU patients ≥18 years with an expected ICU stay of ≥5 days and anticipated tube feeding for ≥3 days. Exclusion criteria were the presence of contraindications for enteral nutrition or placement of an enteral feeding tube, patients receiving enteral nutrition with ≥75% of caloric goal already administered, restrictions in full intestinal support, parenteral nutrition of any kind unless due to enteral nutrition intolerance, a history of allergy or intolerance to study product components (test or control product), nonfunctional gastrointestinal tract, limited care, and participation in another interventional trial during the last month.

### Test products

The active product, Peptamen® AF*,* is a liquid tube-feeding calorie-dense formula (1.5 Kcal/ml) not containing fibers. It has a high whey protein concentration delivering 25% of the energy by proteins, 39% by lipids, and 36% by carbohydrates.

The control product, Isosource® Energy, is a liquid tube-feeding, calorie-dense formula (1.5 Kcal/ml) without fibers, delivering 16% of the energy by protein, 35% by lipids, and 50% by carbohydrates. The exact composition of both formulas is indicated in Additional file 1 (Table S6).

### Feeding protocol

Patients in the trial received tube feeding initiated 0 to 72 h post-ICU admission. They received either Peptamen® AF or Isosource® Energy. The caloric target was 25 Kcal/kg/day body weight (taken from medical records or relatives, or estimated by medical staff) to be reached on the third day after enteral nutrition was started. The quantity was subsequently adjusted to caloric estimates by indirect calorimetry to be performed whenever possible during the first night after study start, 3 days afterwards, at end of nutrition with the study product, and 2 days afterwards if still intubated. The total formula intake was monitored via the Patient Data Management System (PDMS) as described below. Tube feeding could be interrupted for clinical reasons (cardiovascular instability, invasive interventions, reanimation, severe diarrhea, high gastric residual volumes or vomiting, and transportation). The study period on the product lasted a maximum of 10 days. Afterwards, patients were switched to standard enteral nutrition if still required. According to internal nursing guidelines, a fecal collector was administered when diarrhea was present or more than three stool passages occurred per shift, but always at the discretion of the treating physician who also evaluated the presence of contraindications (leuco- or severe thrombopenia, rectal obstruction). Standard physiological parameters were continuously recorded in a PDMS (Centricity Critical Care Clinisoft®; General Electrics, Helsinki, Finland). Study-related additional parameters (diarrhea, material costs based on Additional file 1 (Table S5), and nursing workload) were recorded in the same PDMS using specifically designed case report forms which were later exported to a database provided by the local clinical trial unit (CTU). Due to logistic reasons at the bedside, the nurses did not always record consistency and the number of stools exactly at the same time. In such cases, the closest entries for consistency and stool number were merged off-line. In patients with fecal collectors where the content of the collector was measured only once per day, the frequency and number of individual stool events could not be assessed. Patient-days with fecal collectors were therefore omitted for the characterization of the stool events and the calculation of the Whelan score (Additional file 1: Table S1).

Patients were followed-up daily until 2 days after the study end. An additional follow-up visit was conducted 28 days after randomization. There was independent on-site monitoring provided by the CTU to ensure Good Clinical Practice (GCP) compliance and data quality. Adverse and serious adverse events were recorded according to GCP Guidelines and are reported in Additional file 1 (Table S7–S9).

### Statistics

Prior to the current pilot study, we did not formally assess and quantify diarrhea in our patients. Due to the pilot character of the study, a sample size calculation was therefore not performed. The active group and the control group both had a target sample size of *n* = 45. All randomized patients were included in the primary analysis in the arm to which they were randomized regardless of any protocol violations (intention-to-treat principle). If a fecal collector was administered, the number of stool events could not be counted. As a crude estimate of stool events in these patients, the amount of stool in the fecal collector was divided by the average amount of stool per event from all patient days without the fecal collector in the respective group.

Patient characteristics are presented as median and interquartile range (IQR) and number and percentage of patients for continuous and categorical data, respectively. The two primary outcomes were analyzed by negative binomial (number of diarrhea events) and mixed effects logistic regression (diarrhea-free days) with the treatment group as covariate. For a sensitivity analysis, the regression models were adjusted for baseline diarrhea or for antibiotics prescription, respectively. Secondary endpoints were analyzed by Wilcoxon rank-sum tests (non-normal continuous outcomes), Student *t* tests (normal continuous outcomes), Fisher’s exact test (binary outcomes), Poisson regressions with robust standard errors (count outcomes), and Cox proportional hazard models (time-to-event outcomes). The treatment effect is presented as incidence rate ratio (count outcomes), risk ratio (binary outcomes), mean difference (normal continuous outcomes), Hodges–Lehmann median differences (non-normal continuous outcomes), or hazard ratio (HR; time-to-event outcomes) with 95% confidence interval (CI). Line listings and descriptive statistics were used to analyze adverse event data. In a post-hoc analysis, patients that did or did not experience diarrhea during the course of enteral nutrition were compared. The analysis was adjusted for treatment group, age, sex, and Simplified Acute Physiology Score (SAPS) II admission diagnosis using negative binomial (number of diarrhea events), Poisson regression with robust standard errors (count outcomes), median regression (continuous outcomes) or Cox regression (time-to-event outcomes). Results are reported as incidence rate ratio, median difference, and HR with 95% CI, respectively.

## Results

**Fig. 1:**
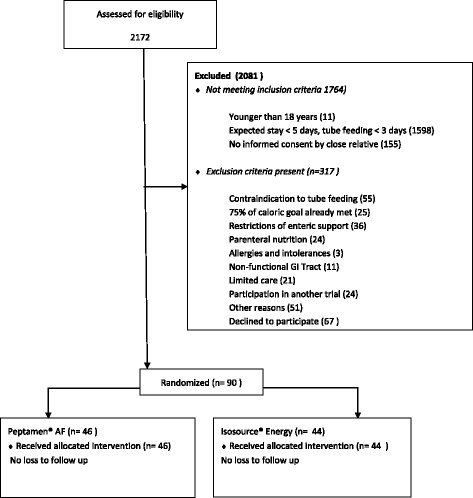
Study CONSORT diagram. *GI* gastrointestinal

Ninety patients were included in the study (intervention group 46, controls 44; Fig. 1). Median age was 63.3 (IQR 51.0–73.2) years and SAPS II was 61.0 (IQR 47.8–74). Five (11%; Peptamen® AF group) and four (9%; Isosource® Energy group) patients, respectively, had diarrhea at study inclusion. Gastric residual volumes did not differ between groups at study inclusion (Table 1). Seventy-three (81%) patients received antibiotics during the course of the study, while 65 (72%) were on antibiotics at study inclusion.

**Table 1:** Patient characteristics

	Peptamen® AF (*n* = 46)		Isosource® Energy (*n* = 44)	
Age, years	*n* = 46	65.3 (52.6–75.3)	*n* = 44	61.6 (48.6–71.3)
Sex, male	*n* = 46	33 (72%)	*n* = 44	28 (64%)
Body mass index, kg/m^2^	*n* = 41	28.8 (25.1–34.2)	*n* = 41	27.8 (23.5–31.5)
APACHE II admission diagnosis	*n* = 44	28.5 (22.3–32.8)	*n* = 42	27.5 (22.0–33.3)
SAPS II admission diagnosis	*n* = 46	60.5 (46.5–74.0)	*n* = 44	61.5 (48.5–75.5)
SOFA	*n* = 45	8.0 (6.0–11.0)	*n* = 44	7.0 (5.0–10.0)
Mechanical ventilation	*n* = 46	43 (94%)	*n* = 44	43 (98%)
Treated with vasoactive drugs	*n* = 46	31 (67%)	*n* = 44	29 (66%)
Diabetes	*n* = 46	13 (28%)	*n* = 44	8 (18%)
Residual gastric volume at study inclusion, ml	*n* = 42	35.0 (10.0–112.5)	*n* = 43	50.0 (20.0–100.0)
Presence of diarrhea at study inclusion	*n* = 46	5 (11%)	*n* = 44	4 (9%)
Blood glucose at study inclusion, mmol/l	*n* = 46	7.8 (6.8–8.9)	*n* = 44	8.3 (6.9–9.4)

Continuous variables are presented as median (interquartile range) and categorical variables as number of patients (%)

*APACHE* Acute Physiology and Chronic Health Evaluation, *SAPS* Simplified Acute Physiology Score, *SOFA* Sequential Organ Failure Assessment

**Fig. 2:**
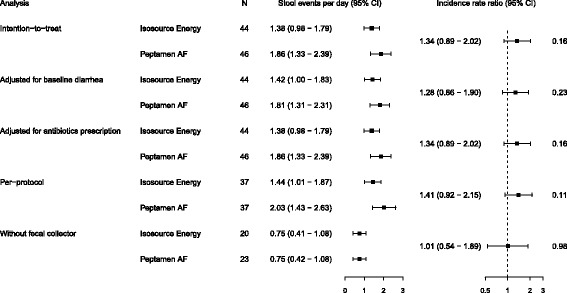
Number of stool events per day in each treatment group. *CI* confidence interval

The time taken to reach the caloric goal and the length of time on study nutrition were not different between groups (Peptamen® AF: 2.2 (0.8–3.7) days and 5.0 (3.6–6.4) days; Isosource® Energy: 2.0 (1.3–2.7) days and 7.0 (5.3–8.7) days; *p* = 0.16 and *p* = 0.26, respectively; Table 2). Sixty patients experienced diarrhea during their ICU stay (Peptamen® AF group: 29 (64%); Isosource® Energy group: 31 (70%); *p* = 0.652). Twenty three (51%) of the Peptamen® group and 24 (55%) patients of the Isosource® group received a fecal collector during the study period because of diarrhea (*p* = 0.83; Table 3). The numbers of diarrhea-free days were 161 (64%) and 196 (68%) for Peptamen® AF and Isosource® Energy, respectively (*p* = 0.65). Stool events per patient ICU days were comparable between groups (Table 4 and Fig. 2 and Additional file 1: Table S1 and S2). In each group, 15 patients received prokinetic drugs (metoclopramide and/or erythromycin). The numbers did also not differ in the per protocol analysis and if only patients without fecal collectors were analyzed. Likewise, the results did not differ between groups when adjusted for baseline diarrhea or for antibiotics prescription (Fig. 2). The material costs were 3.09 (IQR 0.00–7.58) CHF/day/patient. Respective costs per day for patients with diarrhea were CHF 6.31 (0.00–17.11). The median nursing workload in all patients was 15.0 (0.0–27.0) min/day/patient, and for days with diarrhea 40.0 (17.5–74.4) min/patient and for days with fecal collector 20.0 (6.4–52.5) min/patient. Nursing workload and cost for diarrhea care were similar in both groups (Table 3 and Additional file 1: Table S3). Therapeutic intervention scoring system (TISS-76) per day were 46.0 (40.1–54.0) and 46.3 (42.2–50.3) (*p* = 0.83).

**Table 2:** Nutritional intake

	Peptamen® AF (*n* = 46)		Isosource® Energy (*n* = 44)		Treatment effect	*p* value
	*n*	Median (IQR)	*n*	Median (IQR)	Median difference^a^ (95% CI)	
Calorie intake, Kcal/kg/day	41	18.0 (12.5–20.9)	38	19.7 (17.3–23.1)	−2.6 (−5.3 to 0.2)	0.08
Protein intake, g/kg/day	41	1.13 (0.78–1.31)	38	0.80 (0.70–0.94)	0.27 (0.12–0.43)	<0.001
Accumulated caloric deficit during EN/day, Kcal	45	−410 (−984 to −115)	43	−171 (−455 to −4)	−222 (−438 to −45)	0.014
	***n***	**Median (95% CI)**	***n***	**Median (95% CI)**	**HR (95% CI)**	
Length of EN, days	46	5.0 (3.6– to 6.4)	44	7.0 (5.3– to 8.7)	1.35 (0.80–2.26)	0.26
Time to reach the full caloric goal, days	46	2.2 (0.8– to 3.7)	44	2.0 (1.3– to 2.7)	0.70 (0.42–1.16)	0.16
	***n***	**No. of events (pd)**	***n***	**No. of events (pd)**	**Rate ratio (95% CI)**	
Days with parenteral nutrition supplementation	46	5 (253 pd)	44	2 (287 pd)	2.84 (0.28–28.61)	0.38

^a^ Hodges–Lehmann median differences

*CI* confidence interval, *EN* enteral nutrition, *HR* hazard ratio, *IQR* interquartile range, *pd* person days

**Table 3:** Diarrhea-associated events

	Peptamen® AF (*n* = 46)		Isosource® Energy (*n* = 44)		Treatment effect	*p* value
	*n*	*n* (%) with event	*n*	*n* (%) with event	Risk ratio (95% CI)	
Patients that experienced diarrhea during their ICU stay	45	29 (64%)	44	31 (70%)	0.91 (0.68–1.22)	0.65
Patients receiving a fecal collector during their ICU stay	45	23 (51%)	44	24 (55%)	0.94 (0.63–1.39)	0.83
	***n***	**No. of events (pd)**	***n***	**No. of events (pd)**	**Rate ratio (95% CI)**	
Days with interruption of EN due to diarrhea	46	0 (253 pd)	44	2 (287 pd)	nd	0.28
	***n***	**Median (IQR)**	***n***	**Median (IQR)**	**Median difference** ^**a**^ **(95% CI)**	
Total costs of diarrhea per patient/day, CHF	45	3.66 (0.00–8.73)	44	2.60 (0.00–6.39)	0.00 (−0.36 to 2.70)	0.35
Nurse workload per patient/day, min	45	17.0 (0.0–38.5)	44	13.1 (0.0–21.5)	2.5 (−0.7 to 11.8)	0.28

^a^ Hodges–Lehman median differences

*CI* confidence interval, *EN* enteral nutrition, *ICU* intensive care unit, *IQR* interquartile range, *nd* not defined, *pd* person days

**Table 4:** Primary endpoints

	Peptamen® AF (*n* = 46)	Isosource® Energy (*n* = 44)	Rate or risk ratio (95% CI)	*p* value
Stool events				
Intention to treat	542 (253 pd)	415 (287 pd)	1.34 (0.89 to 2.02)	0.16
Adjusted for baseline diarrhea	542 (253 pd)	415 (287 pd)	1.28 (0.86 to 1.90)	0.23
Adjusted for antibiotics prescription	542 (253 pd)	415 (287 pd)	1.34 (0.89 to 2.02)	0.16
Per protocol^a^	527 (237 pd)	401 (273 pd)	1.41 (0.92 to 2.15)	0.11
Diarrhea-free days				
Intention to treat	161 (64%)	196 (68%)	0.82 (0.36 to 1.90)	0.65
Adjusted for baseline diarrhea	161 (64%)	196 (68%)	0.83 (0.38 to 1.82)	0.64
Adjusted for antibiotics prescription	161 (64%)	196 (68%)	0.83 (0.36 to 1.92)	0.66
Per protocol^a^	149 (63%)	185 (68%)	0.82 (0.34 to 1.96)	0.65

^a^ *n* = 37 in both groups

*CI* confidence interval, *pd* person days

**Fig. 3:**
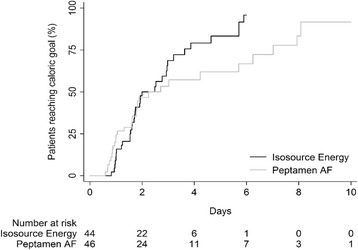
Kaplan-Meier plot for the time to reach the full caloric goal. Patients not reaching the caloric goal are censored (at the end of enteral nutrition)

Some small numerical differences in secondary endpoints between the two groups were identified, but without an overall trend specific to one product, and without clinical relevance (Tables 2, 3, 5, and 6). Both groups received a similar percentage of prescribed calories (median 85% (IQR 71%–95%) for Peptamen®, 90% (84%–96%) for Isosource®®; *p* = 0.07) and reached the caloric goal in equal time (2.2 (0.8–3.7) days for Peptamen®, 2.0 (1.3–2.7) days for Isosource®®; *p* = 0.16; Fig. 3). Median caloric intake did not differ between groups (Peptamen® AF: 18.0 (12.5–20.9) kcal/kg/day; Isosource® Energy: 19.7 (17.3–23.1) kcal/kg/day; *p* = 0.08), with a higher protein intake for the Peptamen® group (1.13 (0.78–1.31) g/kg/day vs 0.80 (0.70–0.94) g/kg/day; *p* < 0.001). Differences were found for the accumulated caloric deficit during enteral nutrition/day, which yielded −410 (−984 to −115) kcal for Peptamen® AF and −171 (−455 to −4) kcal for Isosource® Energy (*p* = 0.014; Table 2).

**Table 5:** Albumin and glucose

	Peptamen® AF (*n* = 46)		Isosource® Energy (*n* = 44)		Treatment effect	*p* value
	*n*	Median (IQR)	*n*	Median (IQR)	Median difference^a^ (95% CI)	
Serum albumin at baseline	39	21.0 (17.0–25.0)	42	21.3 (18.0–24.0)	0.0 (−2.0 to 2.0)	0.93
Serum albumin at treatment end or at ICU discharge	17	21.0 (19.0–26.0)	19	23.0 (18.0–26.0)	0.00 (−3.00 to 3.00)	0.99
	***n***	**No. of events (pd)**	***n***	**No. of events (pd)**	**Rate ratio (95% CI)**	
Number of events outside the 4.5–10 mmol/l glycemic range	46	425 (253 pd)	44	582 (287 pd)	0.83 (0.54–1.28)	0.39

^a^Hodges–Lehmann median differences

*CI* confidence interval, *ICU* intensive care unit, *IQR* interquartile range, *pd* person days

**Table 6:** Length of stay, mechanical ventilation and secondary infections

	Peptamen® AF (*n* = 46)		Isosource® Energy (*n* = 44)		Treatment effect	*p* value
	*n*	No. of events (pd)	*n*	No. of events (pd)	Rate ratio (95% CI)	
Secondary infections	46	19 (253 pd)	44	19 (287 pd)	1.13 (0.70 to 1.84)	0.61
	***n***	**Median time (95% CI)**	***n***	**Median time (95% CI)**	**HR (95% CI)**	
Length of mechanical ventilation, days	46	6.2 (4.8–7.7)	44	7.0 (4.7–9.3)	1.33 (0.83–2.11)	0.23
Length of ICU stay, days	46	7.0 (5.3–8.7)	44	10.0 (6.6–13.4)	1.27 (0.81–2.02)	0.30
Length of hospital stay, days	46	31.0 (27.0–35.0)	44	36.0 (29.9–42.1)	1.01 (0.54–1.89)	0.97

*CI* confidence interval, *HR* hazard ratio, *ICU* intensive care unit, *pd* person days

In the Isosource® group, three patients had positive stool samples for *Clostridium difficile*. There was no noticeable difference regarding adverse events or serious adverse events between the two treatment groups (Additional file 1: Table S7–S9). No ‘certain’ or ‘probable’ product-related events were identified. The length of mechanical ventilation and ICU and hospital stay were similar in both groups (Table 6).

At baseline, patients with diarrhea suffered from higher scores of disease severity (Acute Physiology and Chronic Health Evaluation (APACHE) II and SAPS II; Table 7). A post-hoc analysis adjusted for treatment group, age, sex, and SAPS II score showed that patients with diarrhea remained longer on mechanical ventilation (9.5 (6.0–13.1) days vs. 3.9 (3.2–4.6) days; *p* =0.006) and had longer stays in the ICU (11.0 (8.9–13.1) days vs. 5.0 (3.8–6.2) days; *p* =0.001) (Table 8).

**Table 7:** Baseline characteristics from post-hoc analysis

	Patients with diarrhea (*n* = 60)		Patients without diarrhea (*n* = 30)		*p* value
Receiving Peptamen® AF	*n* = 60	29 (48%)	*n* = 30	17 (57%)	0.51
Age, years	*n* = 60	63.7 (51.0–74.3)	*n* = 30	61.7 (50.5–72.8)	0.71
Sex, male	*n* = 60	41 (68%)	*n* = 30	20 (67%)	1.00
Body mass index, kg/m^2^	*n* = 54	28.1 (23.6–33.8)	*n* = 28	28.2 (23.9–32.6)	0.83
APACHE II score	*n* = 57	30.0 (25.0–36.0)	*n* = 29	26.0 (20.0–30.0)	0.005
SAPS II score	*n* = 60	66.0 (53.5–76.8)	*n* = 30	52.0 (43.8–63.8)	0.003
Diabetes	*n* = 60	12 (20%)	*n* = 30	9 (30%)	0.30
Residual gastric volume at study inclusion, ml	*n* = 58	41.0 (20.0–112.5)	*n* = 27	30.0 (10.0–80.0)	0.19
Presence of diarrhea at study inclusion	*n* = 60	8 (13%)	*n* = 30	1 (3%)	0.26
Blood glucose at study inclusion, mmol/l	*n* = 60	8.2 (7.0–9.4)	*n* = 30	8.0 (6.6–8.9)	0.43

Continuous variables are presented as median (interquartile range) and categorical variables as number of patients (%)

*APACHE* Acute Physiology and Health Evaluation, *SAPS* Simplified Acute Physiology Score

**Table 8:** Post-hoc comparison of patients with and without diarrhea

	Patients with diarrhea (*n* = 60)		Patients without diarrhea (*n* = 30)		Effect of diarrhea	*p* value
	*n*	*n* (%) with event	*n*	*n* (%) with event	Risk ratio (95% CI)	
Death	60	16 (27%)	29	7 (24%)	0.68 (0.26 to 1.78)	0.44
	***n***	**Median (IQR)**	***n***	**Median (IQR)**	**Median difference (95% CI)**	
Maximal abdominal pain per patient/stay^a^	42	0.00 (0.00–3.00)	19	2.00 (0.00–4.00)	−0.8 (−2.9 to 1.4)	0.47
Mean abdominal pain per patient/stay^a^	42	0.00 (0.00–1.00)	19	0.67 (0.00–2.00)	−0.4 (−1.4 to 0.7)	0.49
Accumulated caloric deficit 3 days after start of EN, Kcal	59	−888 (−1828 to −266)	29	−924 (−1521 to −230)	−175 (−813 to 463)	0.59
Accumulated caloric deficit during EN/day, Kcal	59	−268 (−521 to −41)	29	−455 (−1008 to −78)	248 (−67 to 563)	0.12
Percentage of cumulative calories delivered vs. prescribed during EN	59	0.90 (0.81–0.96)	29	0.85 (0.74–0.95)	0.01 (−0.10 to 0.11)	0.91
Serum albumin at baseline, g/L	53	20.0 (16.0–23.0)	28	24.0 (21.0–26.0)	−2.6 (−5.2 to −0.0)	0.050
Serum albumin 3 days after EN start, g/L	43	18.0 (14.0–24.0)	11	23.0 (17.0–25.0)	−0.7 (−4.7 to 3.2)	0.72
Serum albumin at treatment end or at ICU discharge, g/L	23	20.0 (17.0–26.0)	13	24.0 (21.0–25.0)	−0.8 (−4.7 to 3.2)	0.69
	***n***	**No. of events (pd)**	***n***	**No. of events (pd)**	**Rate ratio (95% CI)**	
Stool events	60	911 (418 pd)	30	46 (122 pd)	4.60 (2.98–7.11)	<0.001
Days with interruption of EN due to diarrhea	60	2 (418 pd)	30	0 (122 pd)	nd	
Days with presence of electrolyte and acid-base disturbances	60	350 (418 pd)	30	102 (122 pd)	0.97 (0.88–1.09)	0.64
Days with presence of electrolyte disturbances	60	346 (418 pd)	30	98 (122 pd)	1.01 (0.90–1.13)	0.88
Days with presence of acid-base disturbances	60	42 (418 pd)	30	17 (122 pd)	0.48 (0.27–0.84)	0.010
Changes in intra-abdominal pressure	60	250 (418 pd)	30	53 (122 pd)	1.21 (0.68–2.13)	0.51
Diarrhea due to medication	60	18 (418 pd)	30	0 (122 pd)	nd	
Days with drug interfering with the passage of nutrition	60	150 (418 pd)	30	26 (122 pd)	1.50 (0.76–2.95)	0.25
Number of events outside the 4.5–10 mmol/l glycemic range	60	748 (418 pd)	30	259 (122 pd)	0.58 (0.34–1.01)	0.05
Number of events above the 4.5–10 mmol/l glycemic range	60	738 (418 pd)	30	259 (122 pd)	0.58 (0.33–1.00)	0.05
Number of events below the 4.5–10 mmol/l glycemic range	60	10 (418 pd)	30	0 (122 pd)	nd	
Secondary infections	60	28 (418 pd)	30	10 (122 pd)	0.66 (0.36–1.20)	0.17
Days with mechanical ventilation	60	242 (418 pd)	30	68 (122 pd)	0.98 (0.78–1.24)	0.86
	***n***	**Median time (95% CI)**	***n***	**Median time (95% CI)**	**HR (95% CI)**	
Length of EN, days	60	8.0 (5.9–10.1)	30	4.0 (2.9–5.1)	0.36 (0.20–0.64)	<0.001
Length of ICU stay, days	60	11.0 (8.9–13.1)	30	5.0 (3.8–6.2)	0.40 (0.23–0.71)	0.001
Length of hospital stay, days	60	36.0 (29.6–42.4)	30	31.0 (17.6–44.4)	0.67 (0.33–1.39)	0.29
Length of mechanical ventilation, days	60	9.5 (6.0–13.1)	30	3.9 (3.2–4.6)	0.46 (0.27–0.81)	0.006
Time to reach the full caloric goal, days	60	1.9 (0.9–3.0)	30	2.5 (1.7–3.3)	1.11 (0.59–2.07)	0.75

Effects from an adjusted analysis (for treatment group, age, sex, and Simplified Acute Physiology Score (SAPS) II admission diagnosis) are shown

^a^Patient-assessed, only in non-comatose patients

*CI* confidence interval, *EN* enteral nutrition, *HR* hazard ratio, *ICU* intensive care unit, *IQR* interquartile range, *nd* not defined, *pd* person days

## Discussion

Diarrhea was present during one-third of all ICU days, and roughly two-thirds of our target patient population with a median ICU stay of 8 days experienced diarrhea. The gastrointestinal symptoms were not influenced by the nutritional product. Adjustment for baseline diarrhea (roughly 10% of the patients) and use of antibiotics (61%) did not alter the results. Associated abdominal discomfort was absent or mild-to-moderate in patients in whom it could be assessed, but the nursing workload related to diarrhea was high. The accumulated caloric deficit was <500 Kcal/day during the study period (428 Kcal/day for patients with diarrhea over both groups) but may have been underestimated if malabsorption occurred during periods of diarrhea. The higher caloric deficit in the Peptamen® AF group may be attributed to a higher rate of tube feeding interruptions per nutrition day. Most of the adverse events were judged as unrelated to the study product and the latter was withdrawn in only 3–5% of events.

Previous studies have reported diarrhea incidences ranging from around 15% to over 50% [19–21]. Some of the differences can be explained by a differing case mix and definition of diarrhea. The patients in the present study had several risk factors for diarrhea which were likely to contribute to the observed high diarrhea incidence: use of a hyperosmolar formula in both study groups [22]; a relatively high administration rate of the enteral nutrition [23]; the presence of hypoalbuminemia in all study patients [24, 25]; and frequent use of antibiotics in 77% of patients [26, 27].

The composition of enteral feeding had no effect on diarrhea or feeding tolerance. The higher protein content of the new formula was aimed to facilitate higher protein intake, as recommended by recent guidelines [28]. The relevance of protein intake in ICU patients remains controversial [29]. Fewer surgical ICU patients with deficits in protein intake during enteral nutrition were discharged home [30]. In patients with pneumonia and/or sepsis, higher protein administration decreased mortality [31]. In contrast, a recent trial in patients with acute lung injury was stopped prematurely because administration of higher percentages of estimated protein (76% vs. 54%) and energy needs (85% vs. 55%) increased hospital mortality despite unchanged length of mechanical ventilation or infection rates [32]. An enteral diet rich in medium-chain triglycerides, carnitine, and taurine increased protein and energy intake and reduced feeding intolerance and diarrhea in a single-blind study in ICU patients with overall lower diarrhea incidence than in our study [33]. In our study, the differences in protein intake between the study groups were substantial, but were not associated with differences in diarrhea incidence or any of the secondary clinical outcomes.

There are limited data on resource use for patients with diarrhea, and the few published studies focus on *Clostridium difficile*-associated diarrhea. For example, a multicenter retrospective study reporting adjusted outcomes found longer ICU (8.3 days vs. 6.6 days; *p* < 0.01) and hospital stay (13.2 days vs. 8.5 days; *p* < 0.01) and almost 40% increased total cost for patients with compared to those without *C. difficile*-associated diarrhea [34]. Nursing workload for diarrhea care has not been addressed so far. In our study, with a low incidence of *C. difficile*, the average nursing workload for diarrhea care was more than 3 h/patient, with a moderate increase in material costs. However, also in our study, patients with diarrhea spent more days on mechanical ventilation and in the ICU compared to those without. This finding emphasizes the need for more research on potentially preventable factors associated with diarrhea.

A limitation of our study is the relatively small number of patients and the frequent use of fecal collectors, although this is clinically indicated and potentially able to decrease nursing workload. This prevented a more detailed analysis of stool frequency. Nevertheless, we can give a reliable estimate of stool events. Not using the fecal collector would have interfered with estimates of diarrhea cost and nursing workload as they occur in our unit.

## Conclusions

We found that patients with diarrhea stayed longer in the ICU. This demonstrates that diarrhea is a significant problem in the ICU. While the data of this pilot study do not indicate that modification of the protein and fat content can attenuate the incidence of diarrhea, it does show that a product like Peptamen® AF can effectively deliver a high daily protein amount without overfeeding the ICU patients. More research should be conducted to reduce diarrhea in critically ill ICU long-stayers.

## Additional file

Additional file 1: Supplementary Tables (DOCX 48 kb).

## Abbreviations

APACHE: Acute Physiology and Chronic Health Evaluation
CI: Confidence interval
CTU: Clinical trial unit
GCP: Good Clinical Practice
HR: Hazard ratio
ICU: Intensive care unit
IQR: Interquartile range
PDMS: Patient Data Management System
SAPS: Simplified Acute Physiology Score
