# Supplementary material for: A randomized controlled pilot study to evaluate the effect of an enteral formulation designed to improve gastrointestinal tolerance in the critically ill patient—the SPIRIT trial
Source: Crit Care. 2017 Jun 10;21:140. doi: 10.1186/s13054-017-1730-1 (PMC5466775; doi:10.1186/s13054-017-1730-1)
Supplement: Additional file 1: — Supplementary Tables (DOCX 48 kb). [file 13054_2017_1730_MOESM1_ESM.docx]

A randomized controlled pilot study to evaluate the effect of **an enteral formulation designed to improve gastrointestinal tolerance in the critically ill patient** – the SPIRIT trial

Stephan M. Jakob, Lukas Bütikofer, David Berger, Michael Coslovsky, Jukka Takala

***Electronic supplementary material***

***List of secondary outcomes***

The assessment of the secondary endpoints was done daily over the period of enteral nutrition administration and 2 days after treatment end.

- Incidence and severity of diarrhea
  - Number of patients that experienced diarrhea during their ICU stay
  - Interruption of enteral nutrition due to diarrhea
  - Presence/absence of electrolyte and acid-base disturbances (potassium, chloride, alkalosis/acidosis) secondary to diarrhea
- Presence/absence of other abdominal symptoms
  - If present describe: abdominal distension, pain, absence of bowel sounds.
- Changes in intra-abdominal pressure [33]
- Incidence of nausea, vomiting and regurgitation
- Gastric residual volumes (>500ml)
- Need to use any drug interfering with the passage of nutrition
- Visual analogue scale for abdominal discomfort, assessed in non-comatose patients [34]
- Health Economic burden of Diarrhea during EN administration at ICU
  - Nurse workload for the management of patients with diarrhea during the EN feeding period in ICU
  - Total cost of diarrhea evaluated for each patient who experienced diarrhea (medications, cleaning material, blankets, etc.)
- Time to reach the full caloric goal (25 Kcal/kg/day or as determined for each patient with indirect calorimetry)
- Accumulated daily caloric deficit during 72 hs after start of the study, percentage of cumulative calories delivered vs. prescribed during hospitalization in ICU and accumulated deficit during ICU stay/day (it should be the same)
- Need for parenteral nutrition supplementation during ICU stay (yes/no, economic impact) [35]
- Nutritional assessment: serum albumin at baseline, 3 days after EN start and at treatment end or at ICU discharge (whatever comes first)
- Incidence of secondary infections
- ICU Length of Stay, days on mechanical ventilation during ICU stay (mech. ventilation-free days), hospital LOS
- Comprehensive intestinal microbiota profiling by sequencing-based approaches in stool samples collected right before initiation of EN, 3 days after EN start and at treatment end or at ICU discharge (whatever comes first)
- Determination of diarrhea pathogenicity: infections, fecal cultures for pathogens assessed at diarrhea episodes
- End-point of safety evaluation at baseline, 3 days after EN start and at treatment end or at ICU discharge (whatever comes first), will include: blood cell analysis, hemoglobin, hepatic enzymes and creatinine
- Adverse and serious adverse events from study start until 28 days after randomization

**SPIRIT – Supplemental tables**

**Table S1**: Characteristics of stool events without fecal collector. Patients with a fecal collector over the whole time were excluded.

|  | Peptamen AF (N=41) | Isosource Energy (N=43) | | Rate ratio or median difference* (95% CI) | P value |
| --- | --- | --- | --- | --- | --- |
|  | *no. of events (person-days) or median (IQR)* | |  |  |  |
| Total stool events without fecal collector | 155 (166 pd) | 211 (197 pd) | | 0.87 (0.59 to 1.28) | 0.48 |
| Volume |  |  | |  |  |
| <100g | 54 (166 pd) | 94 (197 pd) | | 0.68 (0.41 to 1.14) | 0.15 |
| 100-200g | 49 (166 pd) | 49 (197 pd) | | 1.19 (0.61 to 2.30) | 0.61 |
| >200g | 31 (166 pd) | 48 (197 pd) | | 0.77 (0.40 to 1.47) | 0.43 |
| missing | 21 (166 pd) | 20 (197 pd) | | 1.25 (0.51 to 3.06) | 0.63 |
| Consistency |  |  | |  |  |
| hard and formed | 20 (166 pd) | 14 (197 pd) | | 1.70 (0.73 to 3.95) | 0.22 |
| soft and formed | 20 (166 pd) | 43 (197 pd) | | 0.55 (0.23 to 1.31) | 0.18 |
| loose and unformed | 59 (166 pd) | 97 (197 pd) | | 0.72 (0.38 to 1.37) | 0.32 |
| liquid | 54 (166 pd) | 54 (197 pd) | | 1.19 (0.62 to 2.27) | 0.61 |
| missing | 2 (166 pd) | 3 (197 pd) | | 0.79 (0.14 to 4.61) | 0.79 |
| Whelan score (per day) | 4.00 (0.67, 8.33) | 3.00 (0.00, 7.80) | | 0.08 (-1.25 to 2.60)* | 0.52 |
| *Hodges-Lehmann median differences |  |  | |  |  |

**Table S2**: Stool volumes for patient-days with fecal collector. Only patients with a fecal collector at any time were included.

|  | Peptamen AF (N=23) | Isosource Energy (N=24) | | Median difference* (95% CI) | P value |
| --- | --- | --- | --- | --- | --- |
|  | *median (IQR)* | |  |  |  |
| Stool volume for patients with fecal collectors (ml per day) | 207 (8, 478) | 79 (23, 252) | | 50 (-33 to 232) | 0.35 |
| *Hodges-Lehmann median differences |  |  | |  |  |

**Table S3**: Nurse workload and costs of diarrhea for patients with diarrhea.

|  | Peptamen AF (N=29) | Isosource Energy (N=31) | | Median difference* (95% CI) | P value |
| --- | --- | --- | --- | --- | --- |
|  | *median (IQR)* | |  |  |  |
| Nurse workload per patient/day, min | 42.5 (27.3, 80.0) | 35.0 (15.0, 70.0) | | 9.4 (-10.7 to 25.8) | 0.39 |
| Total costs of diarrhea per patient/day, CHF | 5.7 (2.0, 16.8) | 6.5 (0.0, 17.4) | | 0.0 (-4.6 to 4.5) | 0.93 |
| *Hodges-Lehmann median differences |  |  | |  |  |

**Table S4**: secondary endpoints

|  | Peptamen AF (N=46) | |  | Isosource Energy (N=44) | | | | | | | | | |  |  | | | | P value | | |
| --- | --- | --- | --- | --- | --- | --- | --- | --- | --- | --- | --- | --- | --- | --- | --- | --- | --- | --- | --- | --- | --- |
|  | *no. of patients, median (IQR)* | | | | | | |  | |  |  | | | *median difference* (95% CI)* | | | | |  | | |
| Gastric residual volume per day, ml | 45 | 28 (12, 107) | | 44 | 43 (27, 111) | | | | | | | | | -11 (-27 to 11) | | | | | 0.22 | | |
| Maximal abdominal pain per patient/stay$ | 28 | 2.00 (0.00, 4.00) | | 33 | 0.00 (0.00, 3.00) | | | | | | | | | 0.00 (0.00 to 1.00) | | | | | 0.43 | | |
| Mean abdominal pain per patient/stay$ | 28 | 0.56 (0.00, 2.00) | | 33 | 0.00 (0.00, 1.00) | | | | | | | | | 0.00 (0.00 to 0.67) | | | | | 0.17 | | |
| Accumulated caloric deficit 3 days after start of EN, Kcal | 45 | -1165 (-1828, -230) | | 43 | -744 (-1341, -266) | | | | | | | | | -343 (-802 to 136) | | | | | 0.15 | | |
| Percentage of cumulative calories delivered vs. prescribed during EN | 45 | 0.85 (0.71, 0.95) | | 43 | 0.90 (0.84, 0.96) | | | | | | | | | -0.05 (-0.11 to 0.00) | | | | | 0.07 | | |
| Serum albumin 3 days after EN start | 24 | 18.0 (15.0, 24.0) | | 30 | 20.0 (15.0, 24.0) | | | | | | | | | -0.5 (-4.0 to 3.0) | | | | | 0.78 | | |
|  | *no. of patients, no. of events (person-days)* | | | | | | | |  | |  | |  | | *rate ratio (95% CI)* | | | |  | | |
| Days with presence of electrolyte and acid-base disturbances | 46 | 220 (253 pd) | | 44 | 232 (287 pd) | | | | | | | | | 1.08 (0.96 to 1.20) | | | | | 0.20 | | |
| Days with presence of electrolyte disturbances | 46 | 217 (253 pd) | | 44 | 227 (287 pd) | | | | | | | | | 1.08 (0.96 to 1.22) | | | | | 0.18 | | |
| Days with presence of acid-base disturbances | 46 | 32 (253 pd) | | 44 | 27 (287 pd) | | | | | | | | | 1.34 (0.79 to 2.28) | | | | | 0.27 | | |
| Changes in intra-abdominal pressure | 46 | 149 (253 pd) | | 44 | 154 (287 pd) | | | | | | | | | 1.10 (0.73 to 1.65) | | | | | 0.66 | | |
| Days with abdominal symptoms | 46 | 22 (253 pd) | | 43 | 14 (284 pd) | | | | | | | | | 1.76 (0.78 to 3.97) | | | | | 0.17 | | |
| Incidence of nausea | 45 | 13 (243 pd) | | 43 | 6 (284 pd) | | | | | | | | | 2.53 (0.72 to 8.90) | | | | | 0.15 | | |
| Incidence of vomiting | 45 | 13 (251 pd) | | 43 | 9 (284 pd) | | | | | | | | | 1.63 (0.49 to 5.46) | | | | | 0.43 | | |
| Incidence of regurgitation | 45 | 13 (251 pd) | | 41 | 16 (280 pd) | | | | | | | | | 0.91 (0.28 to 2.91) | | | | | 0.87 | | |
| Days with abdominal discomfort (VAS>0) | 29 | 32 (174 pd) | | 33 | 33 (216 pd) | | | | | | | | | 1.20 (0.64 to 2.28) | | | | | 0.57 | | |
| Days with presence of gastric residual volumes >500ml | 46 | 8 (253 pd) | | 44 | 4 (287 pd) | | | | | | | | | 2.27 (0.54 to 9.47) | | | | | 0.26 | | |
| Days with fecal collector | 46 | 87 (253 pd) | | 44 | 90 (287 pd) | | | | | | | | | 1.10 (0.67 to 1.79) | | | | | 0.71 | | |
| Diarrhea due to medication | 46 | 10 (253 pd) | | 44 | 8 (287 pd) | | | | | | | | | 1.42 (0.24 to 8.24) | | | | | 0.70 | | |
| Days with drug interfering with the passage of nutrition | 46 | 75 (253 pd) | | 44 | 101 (287 pd) | | | | | | | | | 0.84 (0.51 to 1.38) | | | | | 0.50 | | |
| Number of events above the 4.5-10 mmol/l glycaemic range | 46 | 423 (253 pd) | | 44 | 574 (287 pd) | | | | | | | | | 0.84 (0.54 to 1.29) | | | | | 0.42 | | |
| Number of events below the 4.5-10 mmol/l glycaemic range | 46 | 2 (253 pd) | | 44 | 8 (287 pd) | | | | | | | | | 0.28 (0.06 to 1.34) | | | | | 0.11 | | |
| Need for insulin unit administration during exclusive EN | 46 | 139 (253 pd) | | 44 | 173 (287 pd) | | | | | | | | | 0.91 (0.64 to 1.30) | | | | | 0.61 | | |
| Need for glucose administration during exclusive EN | 46 | 303 (253 pd) | | 44 | 293 (287 pd) | | | | | | | | | 1.17 (0.92 to 1.49) | | | | | 0.19 | | |
| Days with mechanical ventilation | 46 | 130 (253 pd) | | 44 | 180 (287 pd) | | | | | | | | | 0.82 (0.64 to 1.04) | | | | | 0.10 | | |
|  | *no. of patients, median time (95% CI)* | | | | | | |  | |  | |  | | | *hazard ratio (95% CI)* | | | |  | | |
| Length of EN, days | 46 | 5.0 (3.6 to 6.4) | | 44 | 7.0 (5.3 to 8.7) | | | | | | | | | 1.35 (0.80 to 2.26) | | | | | 0.26 | | |
| Time form EN start until fecal collector, days | 46 | 3.0 (1.2 to 4.8) | | 44 | 4.0 (1.5 to 6.5) | | | | | | | | | 1.00 (0.57 to 1.78) | | | | | 0.99 | | |
| *Hodges-Lehmann median differences | | |  |  | |  |  | | | | | | | | | |  | |  | |  |
| $patient-assessed, only in non-comatose patients | | |  |  | |  |  | | | | | | | | |  | |  | |  |  |
| nd: not defined | | |  |  | |  |  | | | | | | | | |  | |  | |  |  |

**Table S5**: Costs of prespecified care sets

Small Size Intim Care Set:

| 10 Intim Wipes | 0.60 CHF |
| --- | --- |
| 1 Tena comfort Maxi- Incontinence Pad | 0.78 CHF |
| Gloves non-sterile (<= 4 pairs) | 0.20 CHF |
| Molinea  1 Disposable Bed Pad | 0.17 CHF |
| Lactacyd – Intimate Washing Lotion (5 ml) | 0.12 CHF |
| Exipial Lipolotion - Moisturising Lotion (5 ml) | 0.09 CHF |
|  |  |
| Total Cost | 1.96 CHF |

Middle Size Intim Care Set:

| 15 Intim Wipes | 0.90 CHF |
| --- | --- |
| 1 Tena Comfort Maxi- Incontinence Pad | 0.78 CHF |
| Gloves non-sterile (4-7 pairs) | 0.30 CHF |
| Molinea  1 Disposable Bed Pad | 0.17 CHF |
| Lactacyd - Intimate Washing Lotion (5 ml) | 0.12 CHF |
| 1 Washable Bed Pad (cost of laundry cleaning) | 2.04 CHF |
| Exipial Lipolotion - Moisturising Lotion (5 ml) | 0.09 CHF |
|  |  |
| Total Cost | 4.50 CHF |

Large Size Intim Care Set:

| 25 Intim Wipes | 1.50 CHF |
| --- | --- |
| 1 Bed Sheet (cost of laundry cleaning) | 6.12 CHF |
| 1 Tena Slip, (Diaper) | 0.89 CHF |
| Gloves non-sterile (>= 8 pairs) | 0.40 CHF |
| Molinea 1 Disposable Bed Pad | 0.17 CHF |
| Lactacyd - Intimate Washing Lotion (5 ml) | 0.12 CHF |
| 1 Washable Bed Pad (cost of laundry cleaning) | 2.04 CHF |
| Exipial Lipolotion - Moisturising Lotion  (5 ml) | 0.09 CHF |
|  |  |
| Total Cost | 11.33 CHF |

External Fecal Collector Application Set:

| Fecal Collector | 11.44 CHF |
| --- | --- |
| Razor | 2.48 CHF |
| Opsite flexi fix (20cm)- Transparent Adhesive Film | 0.47 CHF |
| 3M Cavilion Barrier Film Swab | 2.09 CHF |
| Gloves non-sterile (8 Pairs) | 0.40 CHF |
| Molinea 1 Disposable Bed Pad | 0.17 CHF |
| 1 Disposable Medical Absorbent Pad | 0.26 CHF |
| Exipial Lipolotion - Moisturising Lotion  (5 ml) | 0.09 CHF |
|  |  |
| Total Cost | 17.40 CHF |

External Catheter Flexi-Seal^®^ Application Set:

| Flexi-Seal^®^ Fecal Management System | 339.34 CHF |
| --- | --- |
| Flexi-Seal^®^ Collection Bag | 7.72 CHF |
| Gloves non-sterile (8 Pairs) | 0.40 CHF |
| 1 Disposable Medical Absorbent Pad | 0.26 CHF |
| Zink Cream 1 Tube | 17.69 CHF |
|  |  |
| Total Cost | 365.41 CHF |

Anal Tampon:

| Anal Tampon | 5.18 CHF |
| --- | --- |

**Table S6.** Composition of the two enteral nutrition products.

|  |  | **Isosource® Energy** | **Peptamen® AF** |
| --- | --- | --- | --- |
| Nutrient Profile | Unit | Per 100 mL | Per 100 mL |
| Energy | kcal / kJ | 157 / 661 | 150 / 630 |
| Protein | g | 6.1  (16% kcal) | 9.4  (25% kcal) |
| Carbohydrate | g | 19.3  (49% kcal) | 13.5  (36% kcal) |
| Sugars | g | 1.7 | 1.4 |
| lactose | g | < 0.1 | < 0.2 |
| Fat  of which | g | 6.2  (35% kcal) | 6.5  (39% kcal) |
| saturates | g | 2.0 | 3.8 |
| medium chain triglycerides | g | 1.24 | 3.4 |
| monounsaturates | g | 2.4 | 0.65 |
| polyunsaturates | g | 1.4 | 1.1 |
| omega-3 | g | 0.29 | 0.36 |
| omega-6 | g |  | 0.82 |
| Fibre | g | 0 | 0 |
| Osmolarity | mOsm/l | 387 | 380 |
| Minerals |  |  |  |
| Sodium | mg | 120 | 100 |
| Chloride | mg | 105 | 80 |
| Potassium | mg | 190 | 230 |
| Calcium | mg | 110 | 100 |
| Phosphorus | mg | 90 | 84 |
| Magnesium | mg | 18 | 30 |
| Iron | mg | 1.6 | 1.6 |
| Zinc | mg | 1.5 | 1.5 |
| Copper | μg | 240 | 130 |
| Iodide | μg | 23 | 14.1 |
| Selenium | μg | 10 | 7.1 |
| Manganese | μg | 0.36 | 0.24 |
| Chromium | μg | 15 | 6.0 |
| Molybdenum | μg | 18 | 12.5 |
| Fluoride | mg | 0.21 | 0.11 |
| Vitamins |  |  |  |
| A | μg RE | 170 | 115 |
| Beta-carotene | μg RE | 43 | 20 |
| D | μg | 2.2 | 1.4 |
| E | mg α-TE | 2.6 | 2.3 |
| K | μg | 12 | 7.8 |
| C | mg | 16 | 18 |
| B1 | mg | 0.24 | 0.28 |
| B2 | mg | 0.27 | 0.22 |
| B6 | mg | 0.28 | 0.29 |
| Niacin | mg NE | 2.7 | 4.0 |
| Folic acid | μg | 45 | 36 |
| B12 | μg | 0.58 | 0.45 |
| Pantothenic acid | mg | 0.86 | 0.94 |
| Biotin | μg | 7.0 | 4.2 |
| Other nutriment: Choline | mg | 60 | - |

**Table S7:** Summary of adverse events (AE)

|  | All AE  n=469 | Isosource® Energy n=241 | Peptamen® AF n=228 |
| --- | --- | --- | --- |
| **Severity** | | | |
| mild | 35 (7%) | 19 (8%) | 16 (7%) |
| moderate | 310 (66%) | 156 (65%) | 154 (68%) |
| severe | 80 (17%) | 41 (17%) | 39 (17%) |
| life-threatening | 18 (4%) | 11 (5%) | 7 (3%) |
| death | 24 (5%) | 12 (5%) | 12 (5%) |
| not assessed | 2 (0%) | 2 (1%) | 0 (0%) |
| **Causality / Relationship to study product** | | | |
| certain | 0 (0%) | 0 (0%) | 0 (0%) |
| probable | 0 (0%) | 0 (0%) | 0 (0%) |
| possible | 65 (14%) | 31 (13%) | 34 (15%) |
| unlikely | 80 (17%) | 46 (19%) | 34 (15%) |
| not related | 321 (68%) | 164 (68%) | 157 (69%) |
| not assessable | 0 (0%) | 0 (0%) | 0 (0%) |
| unknown | 3 (1%) | 0 (0%) | 3 (1%) |

Data presented as number of events (%)

**Table S8.** Serious adverse events (SAE).

|  | | All SAE (n=127) | | Isosource® Energy (n=68) | | Peptamen® AF (n=59) | |
| --- | --- | --- | --- | --- | --- | --- | --- |
| **Classification** | | | | | | | |
| Fatal (yes) | | n = 126, 24 (19%) | | n = 67, 12 (18%) | | n = 59, 12 (20%) | |
| Life threatening (yes) | | n = 126, 25 (20%) | | n = 67, 13 (19%) | | n = 59, 12 (20%) | |
| Inpatient hospitalization or prolongation (yes) | | n = 125, 94 (75%) | | n = 67, 51 (76%) | | n = 58, 43 (74%) | |
| Resulted in persistent or significant disability/incapacity (yes) | | n = 118, 7 (6%) | | n = 62, 2 (3%) | | n = 56, 5 (9%) | |
| Congenital anomaly of an off-spring (yes) | | n = 125, 0 (0%) | | n = 67, 0 (0%) | | n = 58, 0 (0%) | |
| Other important medical condition | | n = 124, 3 (2%) | | n = 66, 2 (3%) | | n = 58, 1 (2%) | |
| **Intervention** | | | | | | | |
| Total daily dose | | n = 125, 1164.2 ± 416.0 | | n = 67, 1174.7 ± 424.0 | | n = 58, 1152.1 ± 409.8 | |
| Route | | n = 121, | | n = 65, | | n = 56, | |
| Gastric | | 121 (100%) | | 65 (100%) | | 56 (100%) | |
| **Follow-up** | | | | | | | |
| Action taken | | n = 126, | | n = 67, | | n = 59, | |
| None | | 78 (62%) | | 44 (66%) | | 34 (58%) | |
| Dose not changed | | 15 (12%) | | 9 (13%) | | 6 (10%) | |
| Study product withdrawn | | 6 (5%) | | 2 (3%) | | 4 (7%) | |
| Other | | 9 (7%) | | 2 (3%) | | 7 (12%) | |
| Not applicable | | 18 (14%) | | 10 (15%) | | 8 (14%) | |
| Is the SAE the cause for study discontinuation? (yes) | | n = 125, 13 (10%) | | n = 67, 8 (12%) | | n = 58, 5 (9%) | |
| Code breaking | | n = 124, 124 (100%) | | n = 67, 67 (100%) | | n = 57, 57 (100%) | |
| Relevant concomitant medication / procedures (yes) | | n = 126, 15 (12%) | | n = 67, 11 (16%) | | n = 59, 4 (7%) | |
| Medical condition (yes) | | n = 125, 125 (100%) | | n = 66, 66 (100%) | | n = 59, 59 (100%) | |
| SAE resolved at this moment of time (yes) | | n = 126, 94 (75%) | | n = 67, 47 (70%) | | n = 59, 47 (80%) | |
| **Outcome** | | | | | | |  |
| Outcome | n = 126, | | n = 67, | | n = 59, | |  |
| recovered | 69 (55%) | | 41 (61%) | | 28 (47%) | |  |
| recovered with sequelae | 2 (2%) | | 1 (1%) | | 1 (2%) | |  |
| recovering | 21 (17%) | | 10 (15%) | | 11 (19%) | |  |
| not recovered | 1 (1%) | | 0 (0%) | | 1 (2%) | |  |
| fatal | 25 (20%) | | 12 (18%) | | 13 (22%) | |  |
| unknown | 8 (6%) | | 3 (4%) | | 5 (8%) | |  |
| **Time to event (days)** | | | | | | |  |
| Time until reporting of SAE | n = 126, 3.0 (1.0; 18.0) | | n = 67, 5.0 (1.0; 20.0) | | n = 59, 2.0 (1.0; 15.0) | |  |
| Time until safety involvement | n = 126, 6.5 (2.8; 22.0) | | n = 67, 7.0 (3.0; 26.0) | | n = 59, 5.0 (2.0; 21.0) | |  |
| Time until first notification | n = 126, 5.0 (1.0; 22.0) | | n = 67, 5.0 (1.0; 37.0) | | n = 59, 4.0 (1.0; 19.0) | |  |
| Time until death | n = 24, 0.0 (0.0; 1.0) | | n = 12, 0.0 (0.0; 1.8) | | n = 12, 0.0 (0.0; 0.8) | |  |
| Time until FUP | n = 125, 9.0 (1.0; 33.5) | | n = 67, 10.0 (1.0; 34.0) | | n = 58, 9.0 (1.0; 33.0) | |  |

Continuous variables are presented as mean ± SD or median (IQR), binary variables as number of events (%).

**Table S9.** Other observations related to safety.

|  | Safety population  N=89 | Isosource® Energy  N=44 | Peptamen® AF  N=45 |
| --- | --- | --- | --- |
| **Baseline** | | | |
| Hemoglobin, g/L | n = 88, 100.0 (89.6; 113.5) | n = 44, 99.5 (89.6; 111.6) | n = 44, 100.3 (89.3; 115.4) |
| ASAT, U/L | n = 86, 72.5 (36.5; 180.3) | n = 44, 66.5 (35.5; 295.3) | n = 42, 78.5 (41.0; 142.5) |
| GGT, U/L | n = 82, 54.5 (22.0; 122.3) | n = 43, 55.0 (21.0; 122.0) | n = 39, 48.0 (22.0; 123.0) |
| Creatinine, μmol/L | n = 87, 107.0 (60.0; 178.0) | n = 43, 98.0 (59.0; 190.0) | n = 44, 116.0 (66.0; 173.8) |
| **3 days after EN start** | | | |
| Hemoglobin, g/L | n = 63, 96.5 (89.0; 111.0) | n = 34, 95.3 (89.0; 109.1) | n = 29, 98.0 (88.5; 114.0) |
| ASAT, U/L | n = 61, 66.0 (35.5; 111.0) | n = 33, 75.0 (42.5; 144.0) | n = 28, 54.5 (32.0; 101.5) |
| GGT, U/L | n = 56, 133.8 (63.3; 230.0) | n = 31, 133.5 (64.0; 232.0) | n = 25, 134.0 (59.5; 229.5) |
| Creatinine, μmol/L | n = 66, 76.5 (54.3; 149.0) | n = 35, 77.0 (52.0; 167.0) | n = 31, 73.0 (59.5; 136.0) |
| **At EN end** | | | |
| Hemoglobin, g/L | n = 60, 98.5 (89.0; 108.4) | n = 31, 99.0 (89.0; 105.0) | n = 29, 97.0 (88.8; 110.8) |
| ASAT, U/L | n = 54, 63.0 (37.8; 143.0) | n = 28, 59.0 (37.0; 96.0) | n = 26, 67.0 (47.4; 203.3) |
| GGT, U/L | n = 29, 208.0 (109.5; 302.5) | n = 17, 217.0 (118.5; 374.5) | n = 12, 119.5 (68.3; 239.0) |
| Creatinine, μmol/L | n = 77, 91.0 (56.0; 142.5) | n = 38, 100.0 (50.8; 203.1) | n = 39, 87.0 (56.0; 113.0) |

Data are presented as median (IQR).
